# The association between nonverbal fluency and anomia treatment outcomes in people with post-stroke aphasia

**DOI:** 10.3389/fnhum.2026.1913940

**Published:** 2026-09-03

**Authors:** Deena Schwen Blackett, Victoria A. Diedrichs, Alexandra Zezinka Durfee, Courtney C. Jewell, Stacy M. Harnish

**Affiliations:** 1 School of Communication Sciences and Disorders, College of Health Professions and Sciences, University of Central Florida, Orlando, FL, United States; 2 Department of Speech and Hearing Science, College of Arts and Sciences, The Ohio State University, Columbus, OH, United States; 3 Department of Communicative Sciences and Disorders, College of Communication Arts and Sciences, Michigan State University, East Lansing, MI, United States; 4 Department of Speech-Language Pathology and Audiology, College of Health Professions, Towson University, Towson, MD, United States

**Keywords:** aphasia, executive function, generalization, language, naming treatment

## Abstract

**Introduction:**

Many people with aphasia experience concomitant executive functioning deficits, and the literature supports a role of executive functioning in treatment response. However, the optimal executive functioning assessment for measuring this impact is unclear, as is the extent to which outcomes (e.g., acquisition, generalization) may be impacted differently. The present retrospective study aimed to determine the degree to which nonverbal fluency, one measure of executive functioning with minimal language demand, was associated with naming treatment acquisition, stimulus generalization, and response generalization in people with chronic aphasia. We hypothesized that nonverbal fluency would be positively associated with treatment acquisition and both forms of generalization, based on the existing literature.

**Methods:**

This retrospective study included 13 participants with chronic, post-stroke aphasia from a completed clinical trial. Participants completed 2 weeks of a cued picture-naming treatment. Using linear regression, we examined the association between baseline nonverbal fluency and Tau-*U* treatment effect size for trained (acquisition) and untrained items (response generalization) and percent stimulus generalization (definition naming).

**Results:**

Controlling for aphasia severity, nonverbal fluency was a small but significant correlate of response and stimulus generalization, but not of treatment acquisition.

**Discussion:**

Nonverbal fluency may be associated with generalization, whereby executive functioning abilities may support generalizing aphasia interventions beyond trained items and contexts. However, more research is needed to investigate the potential association between executive functioning and various treatment outcomes as well as its clinical utility.

## 1 Introduction

Executive functioning (EF) is an umbrella term that encompasses meta-cognitive skills used to plan, carry out, or monitor cognitive tasks. There has been considerable debate surrounding the definition and measurement of EF; however, contemporary accounts mostly refer to EF as a collection of dissociable cognitive skills (Baddeley, 2012; Diamond, 2013; Lezak et al., 2004; Miyake et al., 2000; Miyake and Friedman, 2012). Miyake et al. (2000) present evidence for three distinguishable but related components: (1) *inhibition* of dominant/automatic responses, (2) *updating* knowledge with new and relevant details learned from monitoring incoming information, and (3) *shifting* between tasks or mental states.

Numerous studies investigating EF in people with aphasia (PWA) find that acquired language and metacognitive skill deficits are common comorbidities (Murray, 2017; Purdy, 2002). EF deficits likely occur in most PWA, though the impact and extent of deficits vary considerably (Dutta et al., 2023). For example, inhibition deficits may result in an inability to resolve semantic competition during lexical selection (Martin and Allen, 2008), particularly under cognitively demanding conditions (Obermeyer et al., 2020), highlighting the direct influence of EF on language ability. However, EF may also have an indirect influence on language ability since aphasia therapy often involves *updating* knowledge with newly learned information, *switching* between tasks or stimuli, and *inhibiting* incorrect responses.

Moreover, the severity of aphasia and EF deficits may differ (Dutta et al., 2023; Helm-Estabrooks, 2002). This variability in linguistic and cognitive ability may naturally contribute to differences in treatment response, resulting in some individuals making relatively more meaningful improvements while others may experience little benefit. As such, researchers have considered the impact of EF skills on response to aphasia treatments (Conroy and Scowcroft, 2012; Dignam et al., 2017; Fillingham et al., 2006; Gilmore et al., 2019; Goldenberg et al., 1994; Kendall et al., 2014; Lambon Ralph et al., 2010; Nicholas et al., 2011; Rose et al., 2013; Sandberg et al., 2021; Seniow et al., 2009; Simic et al., 2020; van de Sandt-Koenderman et al., 2007; Votruba et al., 2013). A recent scoping review explored the association between EF and aphasia treatment response, reporting mixed findings (Diedrichs et al., 2022). Most studies examining EF analyzed its association with immediate treatment outcomes on targeted items. Across 15 studies examining this association, immediate language treatment acquisition appeared to be associated with or influenced by EF in six (Conroy and Scowcroft, 2012; Dignam et al., 2017; Fillingham et al., 2006; Gilmore et al., 2019; Lambon Ralph et al., 2010; Nicholas et al., 2011). Only five of 15 studies examined the relationship between EF tasks and either stimulus (i.e., to an untrained task) or response (i.e., to untreated items) generalization. Three of these reported positive statistical associations (Dignam et al., 2017; Nicholas et al., 2011; Simic et al., 2020) and the remaining two reported similar anecdotal findings (Kendall et al., 2014; Sandberg et al., 2021). Despite the inconclusive evidence of EF’s role in treatment response, the scoping review generally supports a role of EF in generalization outcomes. Due to the meta-cognitive nature of EF, such skills may aid PWA in applying treated concepts or strategies to new language targets (Simic et al., 2020) or situations.

Just as EF lacks a unified definition, it also lacks a unified measure. Diedrichs et al. (2022) reported measures of EF employed by the literature, including: (1) subtests from the Cognitive Linguistic Quick Test (CLQT; Gilmore et al., 2019; Helm-Estabrooks, 2001; Nicholas et al., 2011); (2) number of categories completed on the Wisconsin Card-Sorting Test (WCST; (Fillingham et al., 2006; Grant and Berg, 1993); (3) the Trails and Sorting tasks of the Delis-Kaplan EF System (D-KEFS; Delis et al., 2001; Dignam et al., 2017); and (4) a cognitive factor, identified via principal components analysis, reflecting scores on the WCST and other assessments (Lambon Ralph et al., 2010). In addition to these assessment methods, fluency tasks have been used to measure EF. Fluency comprises “the ability to utilize one or more strategies that maximize the production of responses while avoiding response repetition” (Ruff et al., 1994, p. 41). Both verbal and nonverbal fluency tasks are often used as measures of EF, including strategy generation, inhibition, and self-monitoring (Ross et al., 2003). In a verbal fluency task, participants may be asked to name as many semantically (e.g., animals) or phonologically (e.g., words that start with *f*) related items as possible in 1 min (i.e., category and letter fluency, respectively). However, for PWA, verbal fluency tasks are likely confounded by language impairment (Bose et al., 2022). Therefore, nonverbal fluency tasks that minimize language demands, like the Ruff Figural Fluency Test (RFFT; Ruff, 1996) may provide a better controlled equivalent to assess EF among PWA.

On the RFFT, participants are asked to create as many unique designs as possible in 1 min by connecting series of dots. Importantly, the RFFT engages the three dissociable components of EF (Miyake et al., 2000): participants must *update* their knowledge of available designs after each one they draw, *inhibit* previous designs to avoid repetition, and *shift* between the tasks of planning their next design and drawing it. Participants must also update and shift their approach between subtests as distractors and different configurations are introduced. As a result, the RFFT is an efficient and clinically feasible assessment to administer to capture a combination of EF skills. Moreover, the number of unique designs generated has been correlated with other measures of EF in PWA (Murray, 2017). Considering the literature supporting a relationship between other measures of EF and aphasia treatment outcomes (Conroy and Scowcroft, 2012; Dignam et al., 2017; Fillingham et al., 2006; Gilmore et al., 2019; Lambon Ralph et al., 2010; Nicholas et al., 2011; Simic et al., 2020), performance on the RFFT may similarly demonstrate a relationship with aphasia treatment response. Although the RFFT has been employed in aphasia research previously (Dutta et al., 2023, 2024; Murray, 2017), it has not been used to predict aphasia treatment outcomes. Therefore, because the RFFT is a brief, nonverbal assessment that engages multiple EF components while minimizing language demands, it may offer a practical alternative to existing measures for predicting aphasia treatment response.

The present study aimed to determine the degree to which performance on the RFFT is associated with naming treatment acquisition and generalization to untreated words and contexts among PWA. We hypothesized that RFFT scores would be positively associated with treatment acquisition and generalization because the underlying EF components may support word retrieval in the context of naming therapy, such as inhibition supporting resolution of semantic competition (Martin and Allen, 2008) and shifting ability supporting response generalization to untrained targets (Simic et al., 2020). Shifting, inhibiting, and working memory updating may all be involved in applying trained skills to new contexts to support stimulus generalization, although this has seldom been examined in the existing literature (Nicholas et al., 2011).

## 2 Methods

We conducted a retrospective study on data collected during a 2019 clinical trial. The trial was approved by The Ohio State University Institutional Review Board, and participants provided informed written consent to participate (IRB protocol: 2013B0484). All assessments and treatment were administered by trained speech-language pathologists (SLPs) or trained graduate student clinicians.

### 2.1 Participants

Thirteen PWA (11 male, 2 female) were included in this study (see Table 1). Participants were native English speakers and at least 6 months post left-hemisphere stroke. On average, participants were 60.5 ± 14.4 years old (Range: 36–78) with 15.2 ± 2.6 years of education (Range: 12–20) and 51 ± 56.5 months post-stroke (Range: 6–178). Participants were excluded if they had severe depression as indicated on the Beck Depression Inventory (Beck et al., 1996), uncorrected visual or hearing impairment, or other neurological disorder affecting the brain besides stroke.

**Table 1:** Participant demographics.

Participant	Age (years)	Gender	Race/Ethnicity	Education (years)	MPO	Lesion location	Stroke mechanism
P1	72	M	W	20	178	L frontal, parietal, occipital	Ischemic
P2	36	M	W	17	33	L MCA	Ischemic (thrombus)
P3	62	M	W	12	14	L MCA	Ischemic
P4	36	M	W	18	47	L MCA	Ischemic
P5	40	F	B	14	12	L MCA	Ischemic
P6	67	M	W	14	21	L MCA	Ischemic (embolic)
P7	63	M	W	17	154	L MCA	Unknown (infarct)
P8	70	M	W	12	6	L MCA	Ischemic (embolic)
P9	78	F	W	12	74	L MCA	Ischemic (embolic)
P10	69	M	W	16	27	L MCA	Unknown (infarct)
P11	72	M	W	13	81	L MCA	Ischemic
P12	67	M	B	17	11	L MCA	Ischemic
P13	55	M	W	16	9	L MCA	Mycotic aneurysmal subarachnoid, intraventricular, and intraparenchymal hemorrhage

M, male; F, female; W, White; B, Black/African American; MPO, months post onset of stroke; L, left; MCA, middle cerebral artery.

Aphasia diagnoses were confirmed via the Western Aphasia Battery-Revised (WAB-R) by an Aphasia Quotient (AQ) of less than 93.8 (Kertesz, 2006). Participants had significant but not profound anomia as determined by a score of 4-44^1^ on the Boston Naming Test (Kaplan et al., 2001) and relatively preserved auditory comprehension abilities to ensure comprehension of all task instructions (i.e., ≤2 standard deviations from the norming sample mean on the WAB-R Auditory Verbal Comprehension Score). Participants with severe-profound apraxia of speech were excluded based on ratings from two certified SLPs otherwise unaffiliated with the study on a series of speech tasks (Dabul, 2000; Goodglass et al., 2000). A third SLP resolved disagreements. Participants also completed an intake questionnaire (MacWhinney et al., 2011), including questions about handedness and hemiparesis. See Table 2 for participant language profiles and handedness.

**Table 2:** Participant language and nonverbal fluency scores.

Participant	WAB-R AQ	WAB-R AVC	BNT	Semantic fluency (animals)	RFFT total unique designs	Hand used on RFFT	Pre-stroke handedness	Right arm strength self-report ratings
P1	60.0	164	14	2	22	Left	Right	Very weak
P2	71.8	166	33	9	35	Right	Right	A little weak
P3	57.4	142	5	0	34	Right	Right	A little weak
P4	54.8	112	19	5	57	Left	Left	Very weak
P5	63.8	154	9	4	55	Left	Right	Very weak
P6	76.5	169	38	15	88	Left	Right	Very weak
P7	93.5	189	41	12	39	Right	Right	Good strength
P8	48.1	153	6	7	59	Left	Right	Very weak
P9	39.0	122	10	0	33	Right	Right	Good strength
P10	54.1	141	6	4	44	Right	Right	Good strength
P11	45.0	150	14	7	33	Left	Right	Very weak
P12	78.2	180	44	9	35	Left	Right	Very weak
P13	71.6	146	13	8	39	Right	Right	A little weak

WAB-R AQ scores are out of a total weighted score of 100, where 0–25 = very severe; 26–50 = severe; 51–75 = moderate; and ≥76 = mild aphasia. WAB-R AVC scores are out of a total raw score of 200. The BNT has a total raw score of 60. Semantic fluency (animals) scores are raw scores from the Word Fluency subtest of the WAB-R (max score: 20). RFFT unique designs has a theoretical range of 0–175 and normal ranges among adults without aphasia or other neurological disorders have varied from 47.6 to 134.3, depending on age and years of education (Ruff et al., 1987). Arm strength ratings were reported on a scale including good strength, a little weak, very weak, and paralyzed. All participants rated left arm strength as good. BNT, Boston Naming Test; RFFT, Ruff Figural Fluency Test; WAB-R AQ, Western Aphasia Battery-Revised Aphasia Quotient; WAB-R AVC, Western Aphasia Battery-Revised Auditory Verbal Comprehension.

### 2.2 Assessment of executive function

Prior to treatment, participants completed the RFFT, a measure of nonverbal fluency with favorable psychometric properties (Ross, 2014; Ruff, 1996). The RFFT comprises five subtests completed on paper. On each page, there are several rows of squares. In Parts 1–3, each square contains five dots arranged concentrically. Part 2 includes diamond figures within the squares as distractors and Part 3 includes extraneous printed lines as more complex distractors. Parts 4 and 5 do not include distractors but present new arrangements of the five dots. Participants were instructed to draw at least one straight line connecting at least two dots in each box, creating as many unique configurations as possible in 1 min. Participants were given the option to use their unaffected hand. Due to potential motor impairments or hemiparesis, in the event that a line drawn between two points was not straight, authors discussed qualities including line curvature, approximation to the intended dot target(s), and degree of motor difficulty in the participant’s affected or compensatory hand to come to consensus regarding its acceptance as a permissible drawing. We accepted close approximations judged not to be true rule violations in an effort to limit the impact of any motor impairments. Seven participants used their left hand to complete the RFFT, including one who was left-handed before their stroke (see Table 2). Number of unique designs on the RFFT was used in our analyses and is measured by subtracting the number of perseverated designs from the total number of designs produced. A higher number of unique designs reflects better EF. See Supplementary material for an analysis comparing the number of unique designs produced by participants using their dominant vs. non-dominant hand, which did not reveal a significant difference.

### 2.3 Treatment and treatment outcome

#### 2.3.1 Word selection

To select individualized stimuli lists, participants attempted to name over 500 pictures, which were presented in a pseudorandomized order on two separate days prior to treatment. Words named incorrectly across both administrations were prioritized for generating stimuli lists. When participants did not name enough words incorrectly twice, words that were named incorrectly once were selected and evenly distributed across the two lists. Twenty words were selected as treatment targets, and 20 additional words were selected to measure generalization to untreated items. Word lists were matched for word frequency, number of syllables, and number of living/nonliving items and were from non-overlapping semantic categories.

#### 2.3.2 Cued-picture naming treatment

Participants underwent an in-person cued picture naming treatment (CPNT; Harnish et al., 2014; Harnish and Lundine, 2015) for 2 weeks, 4 days per week (eight sessions). During the treatment, participants attempted to name a black-and-white drawing representing each word in the treated word list eight consecutive times following various cues provided in the same order by the experimenter: (1) independent naming (no cue), (2) naming following an orthographic cue (the written word beneath the picture), (3) naming following a spoken repetition cue, (4) independent naming following a 3-s delay, (5) naming following three semantic cues (spoken and orthographic presentation), (6) naming following a phonological cue (first phoneme spoken by the experimenter), (7) naming following a repetition cue, and (8) independent naming following a 3-s delay (see Supplementary Figure 1).

If the participant was unable to correctly name the item on any trial, the experimenter provided the correct response and asked the participant to repeat it. The treatment session was terminated after all eight presentations for all 20 words were completed regardless of session duration to keep number of teaching episodes consistent across participants. Treatment fidelity for CPNT was assessed in 24% of randomly selected treatment sessions, and fidelity was found to be 99.3%. See Supplementary material for additional detail.

#### 2.3.3 Picture-naming probes and reliability

To measure treatment outcomes, words that were selected for treatment were probed for picture naming at baseline, the start of each treatment session, post-treatment, and a 3-month follow-up visit. Participants had at least three baseline sessions wherein treated and untreated picture naming word lists were probed. If there was an ascending trend in performance on the treated word list across baseline sessions, then additional sessions were added until there was no ascending trend in three consecutive sessions prior to initiating treatment.

The treated picture naming word list was used to measure treatment acquisition, whereas the untreated picture naming word list was used to measure response generalization. Intra- and inter-rater reliability for picture-naming probes was assessed in 17% of randomly selected sessions and was found to be 97.8 and 97.2%, respectively. See Supplementary material for additional details.

#### 2.3.4 Treatment acquisition and response generalization outcome

Single-subject treatment effects based on picture-naming probes were measured using Tau-*U* (Parker et al., 2011) to control for baseline trends via the online calculator at singlecaseresearch.org (Vannest et al., 2016). Tau-*U* measures effect size as nonoverlap between two phases (i.e., baseline and intervention phases). From the nonoverlapping data, Tau-*U* corrects for baseline trend via subtraction and accounts for positive treatment phase via addition, making the statistic ideal for single-case experimental design.

#### 2.3.5 Stimulus generalization outcome: definition-naming probes

The same treated items that were used on the picture-naming probes were used on definition-naming probes to examine stimulus generalization of the visual picture naming treatment to an auditory naming-to-definition task. Definition-naming probes were administered twice at baseline and once at post-treatment and the 3-month follow-up. Definitions were audio recorded by a female speaker using a neutral tone at a slightly slower than normal pace. Definitions were piloted on a group of 10 neurotypical control participants to ensure they elicited the target word with at least 90% accuracy. Definitions did not include the root or any derivative of the target word, nor did they include any words that were included in the semantic cues used during CPNT. Participants were given 20 s after the second presentation to name the item. For example, for the target word *ear*, participants would hear an audio recording of the definition “the two parts of the body that you hear with” twice before attempting to name the target word. See treatment outcome scores in Table 3.

**Table 3:** Treatment outcome scores.

Participant	Treated word list Tau-*U*	Untreated word list Tau-*U*	Percent generalization to definition (treated word list)
P1	0.42 (Moderate)	0.17 (Small)	0% (0/10)
P2	0.85 (Very large)	−0.38 (No effect)	40% (4/10)
P3	−0.38 (No effect)	0.21 (Moderate)	0% (0/15)
P4	0.79 (Large)	0.79 (Large)	33% (4/12)
P5	1.00 (Very large)	0.75 (Large)	80% (8/10)
P6	1.00 (Very large)	0.79 (Large)	80% (4/5)
P7	0.83 (Very large)	−0.54 (No effect)	100% (3/3)
P8	1.00 (Very large)	0.75 (Large)	40% (4/10)
P9	0.84 (Very large)	0.50 (Moderate)	0% (0/0)
P10	1.00 (Very large)	0.17 (Small)	11% (2/18)
P11	0.75 (Large)	0.63 (Large)	12.5% (1/8)
P12	0.94 (Very large)	0.72 (Large)	100% (2/2)
P13	0.67 (Large)	0.58 (Moderate)	7.14% (1/14)

Per Vannest and Ninci (2015), Tau-*U* effect sizes of <0.20 were considered small; 0.20 to <0.60, moderate; 0.60 to <0.80, large; and ≥0.80, very large. Percent generalization scores in parentheses signify the number of generalized to definition items out of the total items acquired on picture naming probes at post-treatment.

Percent generalization to definition was measured by first identifying items that participants named incorrectly at baseline picture naming (defined by incorrect response on the majority of baseline probes) and definition naming (defined by incorrect response on both baseline probes). Out of all items that participants named incorrectly on both tasks at baseline, the number of items that participants later named correctly on picture naming at post-treatment were considered acquired picture naming items. Percent generalization to definition was then measured by dividing the number of acquired items that were also named correctly on definition naming at post-treatment (numerator) by the number of all acquired items on picture naming (denominator).

#### 2.3.6 Presentation and scoring

All sessions were audio and video recorded. Naming probes and CPNT were administered on a 14- or 15.6-inch Dell Latitude E6540 or 5590 laptop computer via Eprime 2.0 Professional software (Psychology Tools).^2^ Accuracy on probes and treatment items was scored live by the examiner. Video and audio recordings were used to verify scoring as needed. Self-corrections made by participants (within 10 s for picture-naming and 20 s for definition-naming) were scored as correct.

### 2.4 Data analysis

To examine whether nonverbal fluency skills were associated with performance on CPNT in terms of treatment acquisition and generalization, we conducted a series of regression analyses. Number of unique designs on the RFFT was used as our measure of nonverbal fluency. We controlled for aphasia severity in all regression models using the WAB-R AQ, We conducted three regression analyses using IBM SPSS Statistics (Version 31.0.0.0) to examine relationships between the RFFT, WAB-AQ, and the three dependent variables of interest: (1) Tau-*U* of treated picture-naming items, (2) Tau-*U* of untreated picture-naming items, and (3) percent generalization of treated picture-naming items to definition naming.

## 3 Results

### 3.1 Treatment acquisition via treated items (Model 1)

Original inspection of the data revealed one outlier who did not respond to treatment and resulted in a violation of normality when included. We chose to exclude the outlier as opposed to conducting a transformation to maintain interpretability as both approaches yielded similar results. Results of the multiple regression showed that the model including number of unique designs and aphasia severity was not significantly associated with Tau-*U* of treated items, *F*(2, 9) = 2.83 (*p* = 0.111). Of note, the coefficient for number of unique designs was statistically significant (β = 0.006, *p* = 0.042), though this should be interpreted with caution given that the overall model was not significant. See Supplementary material for more information on assumptions, sensitivity analyses, and regression results.

### 3.2 Response generalization via untreated items (Model 2)

A scatterplot of the residuals revealed heteroscedasticity for Tau-*U* of untreated words (see Supplementary Figure 7); therefore, we conducted a weighted-least squares (WLS) regression. There were no outliers identified, and other assumptions were met. Results showed that the model including number of unique designs and aphasia severity was significantly associated with Tau-*U* of untreated items, *F*(2, 10) = 7.89 (*p* = 0.009), with an *R*^2^ of 61.2% and adjusted *R*^2^ of 53.5%. Coefficients of number of unique designs and aphasia severity were also significant (*p* = 0.003 and *p* = 0.048, respectively). The coefficient of the unique designs predictor was in the expected direction: for every additional unique design (better nonverbal fluency), Tau-*U* increased by 0.013 (See Table 4). The coefficient for aphasia severity was small and indicated for every additional point on the WAB-R AQ (less severe aphasia), Tau-*U* decreased by 0.011. These statistically significant effects on Tau-*U* may not be practically significant given a Tau-*U* effect size of 0.20 is considered small (Vannest and Ninci, 2015). See Supplementary Figures 8, 9 for partial regression plots.

**Table 4:** Coefficient table for weighted least-squares regressions.

Model predictors	*B*	*SE*	*p-*value	*CI*
Model 2 (response generalization)				
Intercept	0.523	0.224	.042	[0.023, 1.022]
Unique designs^*^	0.013	0.003	.003	[0.006, 0.020]
Aphasia severity^*^	−0.011	0.005	.048	[−0.021, −0.0001]
Model 3 (stimulus generalization)				
Intercept	−85.284	27.831	.012	[−147.295, −23.274]
Unique designs^*^	0.851	0.379	.049	[0.006, 1.695]
Aphasia severity^*^	1.373	0.464	.014	[0.339, 2.408]

Model 2 was the weighted least-squares regression predicting Tau-*U* for untreated items. Model 3 was the weighted least-squares regression prediction percent generalization to definition naming. *B*, unstandardized coefficient; *SE*, standard error of coefficient; *CI*, confidence interval; ^*^significant at *p* < 0.05.

### 3.3 Stimulus generalization via naming to definition (Model 3)

A scatterplot of the residuals for percent generalization to definition naming again revealed heteroscedasticity (see Supplementary Figure 10), so we conducted a WLS regression. There were no outliers identified, and all other assumptions were met. Results showed that the model including unique designs and aphasia severity was significantly associated with percent generalization of treated items to definition naming, *F*(2, 10) = 9.51 (*p* = 0.005), with an *R*^2^ of 65.5% and adjusted *R*^2^ of 58.7%. Coefficients of number of unique designs and aphasia severity were both significant as well (*p* = 0.049 and *p* = 0.014, respectively). The coefficient of the unique designs predictor was in the expected direction: for every additional unique design (better nonverbal fluency), percent stimulus generalization increased by 0.851% (see Table 4). The coefficient for aphasia severity indicated for every additional point on the WAB-R AQ (less severe aphasia), percent stimulus generalization increased by 1.37%. See Supplementary Figures 11, 12 for partial regression plots.

## 4 Discussion

To test the hypothesis that post-stroke nonverbal EF skills of PWA, independent of severity, inform response to CPNT, we conducted three regression analyses including number of unique designs on the RFFT (EF ability) and WAB-R AQ (aphasia severity) as predictors of treatment acquisition, response generalization, and stimulus generalization. Results showed that EF ability was significantly associated with response and stimulus generalization following anomia treatment but not treatment acquisition.

### 4.1 Acquisition

In contrast to our hypothesis, our first model examining acquisition of treated items was not significant. There are multiple possible explanations for this finding. First, different cognitive abilities may be recruited at different stages of recovery for different purposes (Simic et al., 2020). EF skills are crucial for engaging with new information (Diamond, 2013) and therefore may be more likely to support generalization as well as very early acquisition, when the content is still new. A participant’s response to repeated training and the development of automaticity in CPNT may no longer be supported by EF once the content is familiar, perhaps instead relying on other skills, such as semantic memory, which we did not assess. This finding and this explanation are consistent with past work revealing that specific measures of EF (i.e., reflecting shifting, working memory updating, and common executive control) were not associated with immediate acquisition (e.g., Simic et al., 2020).

However, other studies have implicated EF skills in immediate post-treatment outcomes. For example, the D-KEFS Trails and Sorting tasks both correlated with immediate outcomes for items treated in an intensive comprehensive aphasia program (ICAP; Dignam et al., 2017), which included semantic feature analysis (SFA) and phonological components analysis (PCA) protocols. Gilmore et al. (2019) conducted a principal components analysis to derive non-linguistic cognitive components used to predict treatment outcomes. EF was characterized by a component onto which the symbol trails, mazes, and design generation subtests of the CLQT, as well as other tasks, loaded most prominently. This component was a significant predictor of immediate post-treatment gains following a typicality-based SFA treatment. Importantly, the treatments used in both studies likely included strategy and goal-oriented behavior related to the generation and integration of the lexical features of target words, unlike the CPNT used in the current study. However, Simic et al. (2020) also used PCA and did not find evidence of their EF measures predicting immediate treatment outcomes. Notably, their EF measures included a trails task like the ones used in the other studies (Dignam et al., 2017; Gilmore et al., 2019), but Simic et al. (2020) may have been underpowered with a sample of 10 to identify such a relationship. Taken together, heterogeneity in methods and interventions, in conjunction with the task impurity problem (Snyder et al., 2015; more below), make it difficult to draw broad conclusions about the role of EF in acquisition, but inconsistent findings among similar methodologies suggest additional research is needed to examine the role of EF in acquisition.

A lack of model significance in our study may also be related to limited sample variability in Tau-*U* for acquired treated items (see Treated Word List in Table 3). Despite lack of model significance, the coefficient for the number of unique designs was a statistically significant individual predictor (*p* = 0.042), suggesting that perhaps a significant association would emerge with a larger sample. A post-hoc power analysis (*pwrss* package; Bulus and Jentschke, 2025) in *R* (R Core Team, 2025) revealed that power for our acquisition model was 58.5% for the observed *R*^2^ (0.386), suggesting that we were indeed underpowered to detect a significant relationship. Future studies would need at least 19 participants to detect a similarly sized significant association with 80% power, if present.

### 4.2 Generalization

We found a significant relationship between nonverbal EF and response generalization, aligning with previous research on anomia outcomes and measures of EF including shifting ability (Simic et al., 2020) and sorting (Dignam et al., 2017). As suggested by Martin and Allen (2008), perhaps more intact EF skills improved resolution of semantic competition during lexical retrieval for less rehearsed untrained items. Future studies should conduct error analyses of untrained items from pre- to post-treatment to more directly examine this hypothesis. Unique designs were also significantly positively associated with stimulus generalization in our study, which is not as frequently examined in the literature. However, some prior research on EF and working memory, a cognitive skill closely tied to EF, does support this finding. Nicholas et al. (2011) found that more preserved EF, based on a design generation task similar to the RFFT and other nonverbal tasks from the CLQT, corresponded to better carryover of trained communication device use in untrained contexts for individuals with severe aphasia. Harnish et al. (2018) also observed that performance on verbal and nonverbal span tasks completed prior to CPNT were highly correlated with definition naming. Some combination of the three dissociable EF skills defined by Miyake et al. (2000) are likely important when applying learned items to untrained contexts, but further research is required to tease apart which specific skills may be most relevant. In our study, the RFFT may have been particularly well-suited to capture the skills most important for generalization, as opposed to acquisition. For example, successfully shifting between and inhibiting competitors may have been more relevant for retrieving untrained words during response generalization, but not as crucial for retrieving trained words that were more rehearsed. Moreover, shifting from processing picture stimuli to auditory definitions and updating the semantic features associated with a word in working memory may have been relevant for stimulus generalization.

Notably, all significant coefficients in our study were relatively small, raising questions of the practical significance of our findings and clinical implications. The unique design coefficient suggests that for every 10 additional unique designs generated, we would expect approximately a 0.10 Tau-*U* increase for response generalization. Given that a Tau-*U* of 0.20 is considered a small effect (Vannest and Ninci, 2015), the relationship between nonverbal fluency and generalization to untrained items appears relatively weak. For stimulus generalization, our results suggested that one would have to produce approximately five additional unique designs to have observed generalization of one additional word out of 20 from picture to definition naming. These findings suggest a supportive but perhaps non-essential influence of EF on generalization. Still, results warrant further investigation to better gauge the clinical significance of these findings.

It is important to note that our untreated word list was probed at every session, potentially reflecting practice effects. However, the significant results of our second model are consistent with our third model examining percent generalization to definition, which could not be influenced by practice effects due to the limited number of definition-naming administrations (probed only three times). Thus, the consistent pattern for these two models may support the relationship between nonverbal fluency and various types of generalization.

### 4.3 Aphasia severity

Surprisingly, less severe aphasia was associated with smaller effects on untrained items in our second model. It is possible that ceiling effects contributed to these results. Although no participants reached the ceiling of correctly naming all 20 items in the untreated word list, participants with milder aphasia, who performed relatively well at baseline, had less room for improvement compared to other participants with more severe aphasia. This discrepancy may not have impacted the treated word list because the treatment facilitated improvement on all words named incorrectly at baseline, even a small number.

Higher scores on the WAB-R were significantly associated with greater percent stimulus generalization in our third model. It would be logical for this effect to be driven by auditory comprehension skills necessary to parse and understand the definition stimuli. To test this, we conducted three post-hoc Pearson correlations, first between WAB-R AQ and WAB-R Auditory Verbal Comprehension (AVC) subscores (*r*(11) = 0.77, 95% CI [0.39, 0.93], *p* = 0.002), second between WAB-R AVC and baseline definition naming accuracy averaged across both word lists (*r*(11) = 0.76, 95% CI [0.37, 0.93], *p* = 0.002), and finally between WAB-R AVC and percent generalization to definition naming (*r*(11) = 0.63, 95% CI [0.12, 0.88], *p* = 0.021). The results suggest that participants with higher WAB-R scores indeed had superior AVC and that higher AVC subscores were positively correlated with both naming to definition performance and generalization to definition naming. Despite our inclusion criteria resulting in a somewhat limited range of auditory comprehension impairment, it is possible that the relationships between EF and treatment outcomes were moderated by aphasia severity (perhaps driven by auditory comprehension in the case of stimulus generalization), but we were unable to explore such interactions due to the small sample.

### 4.4 Limitations and future directions

Several limitations to our study warrant consideration. First, the potential influence of right upper extremity weakness, which is common among PWA, may have impacted results. The RFFT requires drawing, and therefore, can be difficult for those who were right-handed before their stroke. However, the *t*-test comparing unique designs for dominant and non-dominant hand users suggested no significant difference between groups. Another study reported no confounding effects of motor issues on the RFFT and found no correlation between limb apraxia and RFFT scores, suggesting the RFFT can accurately capture nonverbal fluency in PWA (Dutta et al., 2023).

Given our small sample size and the retrospective nature of this investigation, this study was primarily hypothesis-generating and inherently limited in generalizability. Our statistical results should, therefore, be interpreted with caution. Prospective replications in larger, more diverse samples are needed to confirm results. Finally, our use of a single nonverbal fluency assessment with multiple iterations of the same task (the RFFT) may have limited our ability to detect relationships between EF and treatment outcomes. Miyake et al. (2000) point out that it is impossible to fully capture all EF processes with one task, and others have suggested that repeat administrations of the same task may not authentically reflect EF (Calamia et al., 2012; Skidmore et al., 2023). Relatedly, EF tasks like the RFFT, are victim to the task impurity problem (Snyder et al., 2015) making it difficult to isolate EF skills from other processes engaged during task completion (e.g., visuospatial processing, initiation, processing speed). While the RFFT has the advantage of reducing the confound of language for PWA, future work should consider the potential benefits of using multiple measures addressing dissociable skills involved in EF (e.g., Simic et al., 2020) with novel stimuli to predict treatment acquisition, maintenance, and generalization. In doing so, however, it is important to consider downstream clinical feasibility if EF measures do, indeed, have prognostic value. In addition, future studies using the RFFT could document self-monitoring and strategy use during both the RFFT and treatment to more thoroughly evaluate the degree to which EF skills were used during task completion.

The current study provides additional evidence aligning with previous work (Simic et al., 2020) that baseline EF skills may be a helpful clinical prognostic indicator in aphasia therapy response and that the RFFT, specifically, may be a practical tool due to its brevity and non-linguistic nature. Notably, nonverbal fluency performance on the RFFT did not always correspond with verbal semantic fluency performance on the WAB-R, shown in Table 2 for comparison. In several cases, nonverbal fluency performance exceeded verbal fluency performance. This pattern illustrates that individuals with substantial language impairment may demonstrate relatively spared nonverbal executive functioning, supporting the potential clinical utility of the RFFT. Such a tool could facilitate identification of individuals with relatively weaker EF skills as potential candidates for infusing EF training into traditional aphasia therapy tasks. A recent systematic review (Culicetto et al., 2025) reported improved post-treatment language performance when EF training was included in isolation or in combination with traditional language-based approaches during intervention. Some of the included studies compared interventions with and without EF training and reported significant improvement in therapy outcome measures over and above intervention without concerted EF training. It remains to be seen how EF-infused training impacts aphasia intervention outcomes in those with relatively intact versus compromised EF post-stroke or if language-specific as opposed to domain-general EF training is associated with better aphasia therapy outcomes, such as previous work has demonstrated with attention (i.e., Language-Specific Attention Training > Direct Attention Training; Peach et al., 2019).

## 5 Conclusion

While behavioral factors, such as aphasia severity (Kristinsson et al., 2023) and structural factors, such as lesion size, location, and white matter integrity (Meier et al., 2019), can account for variance in treatment response, their predictive power is limited. Therefore, it is of clinical interest to identify additional factors that can more robustly explain variability in language therapy response among PWA. Such insight can help individualize care and make efficient use of limited therapy time by selecting interventions that would provide the most benefit. Overall, these results suggest that the domain-general EF skills involved in figural fluency, like inhibition, shifting, and updating working memory, may play a role in response to CPNT for PWA. The small effect sizes in our results suggest that having stronger EF skills may not critically inform response to CPNT but rather provide a small advantage to help some PWA benefit from the therapy. Although further research is required before making intervention recommendations, it may be the case that offering supplementary strategy training to PWA participating in CPNT on an as-needed basis may be preferable to dedicating limited therapy time to explicit training of EF skills.

## Data Availability

The original contributions presented in the study are included in the article/supplementary material. Further inquiries can be directed to the corresponding author/s.

## Footnotes

1 These thresholds were selected based on past clinical research experience on naming recovery with PWA (Harnish et al., 2018).

2
www.pstnet.com
