# Supplementary material for: The association between nonverbal fluency and anomia treatment outcomes in people with post-stroke aphasia
Source: Front Hum Neurosci. 2026 Sep 3;20:1913940. doi: 10.3389/fnhum.2026.1913940 (PMC13595563; doi:10.3389/fnhum.2026.1913940)
Supplement: Supplementary file 1 [file data_sheet_1.docx]

Supplementary Material

Contents:

1. Test for Hand Dominance Difference for Ruff Figural Fluency Test Performance
2. Figure of Cued Picture Naming Treatment (CPNT)
3. Treatment Fidelity Details
4. Picture Naming Measurement Reliability Details
5. Assumptions and Sensitivity Analyses for Model Examining Treated Items
6. Plots for Model 2 Examining Response Generalization via Untreated Items
7. Plots for Model 3 Examining Stimulus Generalization via Percent Generalization of Treated Items to Definition Naming

# 1 Test for Hand Dominance Difference for Ruff Figural Fluency Test Performance

Considering some participants used their dominant hand to complete the Ruff Figural Fluency Test (RFFT), while others used their non-dominant hand due to hemiparesis or hemiplegia, we conducted a *t*-test and checked assumptions in base R (R Core Team, 2025) to compare the number of unique designs in the two groups. The split sample was very small (*n* = 7 dominant hand users, *n* = 6 non-dominant hand users) and although the number of unique designs appeared to be relatively normally distributed for dominant [*W*(*7)* = 0.83, *p* = 0.07] and non-dominant hand users [*W*(*6)* = 0.93, *p* = 0.62], there was evidence of heteroskedasticity between the two groups, based on Levene’s test [*F(*1,11) = 5.95, *p* = .03]. Therefore, Welch’s two sample *t*-test was utilized due to the unequal variances between the groups, which revealed that the number of unique designs produced by dominant hand users (*M* = 40.14, *SD* = 8.34) was not statistically significantly different from non-dominant hand users (*M* = 48.67, *SD* = 23.82), *t*(6) = 0.83, *p* = .436.

# 2 Figure of Cued Picture Naming Treatment (CPNT; Harnish et al., 2014; Harnish & Lundine, 2015)

**Supplementary Figure 1.** CPNT treatment protocol depicting each trial of the protocol for a single item

# 3 Treatment Fidelity Details

Treatment fidelity for CPNT was assessed in 24% of randomly selected treatment sessions using a random number generator. Fidelity was calculated as the total percentage of instances in which the correct picture, cue, or model was provided out of the number of opportunities by a laboratory member who did not conduct the treatment session. For pictures and cues, the number of opportunities was typically 400 per session (eight presentations for each of 20 words), whereas the number of opportunities for modeling was variable and dependent on the number of incorrect responses produced by the participant. Fidelity was found to be 99.3%.

# 4 Picture Naming Measurement Reliability Details

Intra- and inter-rater reliability for our picture-naming probes was assessed in 17% of randomly selected sessions using a random number generator. First, the absolute difference between all intra- or inter-rater scores was calculated (i.e., number of words with different scores from original to re-score). Then, this absolute difference was subtracted from the total number of words across all probes scored for reliability. The resulting value was divided by the total number of words. Intra-rater reliability was 97.8% and inter-rater reliability was 97.2%.

# 5 Assumptions and Sensitivity Analyses for Model 1 Predicting Treatment Acquisition via Treated Items

Upon data visualization, there was an extreme outlier (P3) who was a non-responder to the cued picture naming treatment and had a negative Tau-*U* for treated items. This participant was the only one of the 13 participants to demonstrate no treatment effect. Inclusion of this participant also resulted in a violation of normality for Tau-*U* of treated items. See Supplementary Figures 1 and 2 for visualization of this outlier and Supplementary Figures 3 and 4 for visualization of the data once the outlier was excluded. A square transformation of Tau-*U* for treated items also resolved violations of normality when including all participants, and outliers no longer emerged. There was no evidence of heteroscedasticity or multicollinearity. The Pearson correlation between number of unique designs on the RFFT and the WAB-AQ was .142 (*p* = .321) including all participants. Sensitivity analyses (multiple regression analyses) revealed that a model including all participants and using a square transformation and a model excluding the outlier yielded similar results. We reported the model with raw data excluding the outlier in the main text of the article to aid in interpretation. See outputs for both analyses below in Sections 5.1 and 5.2.

**Supplementary Figure 1.** Histogram of All Participants for Tau-*U* Trained

**Supplementary Figure 2.** Scatterplot of Residuals and Predicted Values for Tau-*U* Trained Revealing Outlier

**Supplementary Figure 3.** Histogram of Participants After Excluding Nonresponder Outlier for Tau-*U* Trained

**Supplementary Figure 4.** Scatterplot of Participants After Excluding Nonresponder Outlier for Tau-*U* Trained

## 5.1 Square Transformation Including Outlier

Regression model: **Trained Tau-*U* Squared** ~ Aphasia Severity (WAB-AQ) + Unique Designs

R^2^ = .389; Adjusted R^2^ = .267

**Supplementary Table 1.** Multiple Regression Table Trained Tau-*U* Squared

|  | Sum of Squares | Degrees of freedom | Mean Square | *F* | *p*-value |
| --- | --- | --- | --- | --- | --- |
| Regression | 0.414 | 2 | 0.207 | 3.189 | .085 |
| Residual | 0.649 | 10 | 0.065 | - | - |
| Total | 1.063 | 12 | - | - | - |

**Supplementary Table 2.** Coefficient Table Trained Tau-*U* Squared

| Model | *B* | *SE* | *p-*value | *CI* |
| --- | --- | --- | --- | --- |
| Intercept | 0.201 | 0.343 | .571 | -0.564 – 0.966 |
| Unique Designs* | 0.011 | 0.004 | .032 | 0.001 – 0.021 |
| Aphasia Severity | 0.0001 | 0.005 | .978 | -0.011 – 0.011 |
| Note. *B* = unstandardized coefficient; *SE* = standard error of coefficient; *CI* = confidence interval; *significant at *p* < .05 | | | | |

## 5.2 Raw Data Excluding Outlier (Reported in Main Text)

Regression model: **Trained Tau-*U*** ~ Aphasia Severity (WAB-AQ) + Unique Designs

R^2^ = .386; Adjusted R^2^ = .250

**Supplementary Table 3.** Multiple Regression Table Trained Tau-*U* Excluding Outlier

|  | Sum of Squares | Degrees of freedom | Mean Square | *F* | *p*-value |
| --- | --- | --- | --- | --- | --- |
| Regression | 0.127 | 2 | 0.063 | 2.829 | .111 |
| Residual | 0.202 | 9 | 0.022 | - | - |
| Total | 0.328 | 11 | - | - | - |

**Supplementary Table 4.** Coefficient Table Trained Tau-*U* Excluding Outlier

| Model | *B* | *SE* | *p-*value | *CI* |
| --- | --- | --- | --- | --- |
| Intercept* | 0.582 | 0.207 | .020 | 0.114 – 1.050 |
| Unique Designs* | 0.006 | 0.003 | .042 | 0.0003 – 0.012 |
| Aphasia Severity | -0.0003 | 0.003 | .924 | -0.007 – 0.006 |
| Note. *B* = unstandardized coefficient; *SE* = standard error of coefficient; *CI* = confidence interval; *significant at *p* < .05 | | | | |

**Supplementary Figure 5.** Partial Regression Plot (RFFT Number of Unique Designs) for Tau-*U* Trained with Regression Line (y = .006x) – Coefficient Significant but Model Not Significant

**Supplementary Figure 6.** Partial Regression Plot (WAB-AQ) for Tau-*U* Trained with Regression Line (y = -.0003x) – Coefficient Not Significant and Model Not Significant

# 6 Plots for Model 2 Examining Response Generalization via Untreated Items

**Supplementary Figure 7.** Residuals vs. Predicted Values for Tau-*U* Untrained Model Showing Heteroscedasticity

**Supplementary Figure 8.** Partial Regression Plot (RFFT Number of Unique Designs) for Tau-*U* Untrained with Regression Line (y = .013x) – Significant Coefficient and Significant Model

**Supplementary Figure 9.** Partial Regression Plot (WAB-AQ) for Tau-*U* Untrained with Regression Line (y = -0.011x) – Significant Coefficient and Significant Model

# 7 Plots for Model 3 Examining Stimulus Generalization via Percent Generalization of Treated Items to Definition Naming

**Supplementary Figure 10.** Residuals vs. Predicted Values for Percent Stimulus Generalization Model Showing Heteroscedasticity

**Supplementary Figure 11.** Partial Regression Plot (RFFT Number of Unique Designs) for Percent Generalization with Regression Line (y = .851x) – Significant Coefficient and Significant Model

**Supplementary Figure 12.** Partial Regression Plot (WAB-AQ) for Percent Generalization with Regression Line (y = 1.373x) – Significant Coefficient and Significant Model
